# Effect of *Hibiscus sabdariffa* L. on the Metabolism of Arachidonic Acid in the Isolated Kidney of a Rat Model of Metabolic Syndrome

**DOI:** 10.3390/ijms241814209

**Published:** 2023-09-18

**Authors:** Israel Pérez-Torres, María Elena Soto, Linaloe Manzano-Pech, Eulises Díaz-Díaz, Raúl Martínez-Memije, Juan Carlos Torres-Narváez, Verónica Guarner-Lans, Vicente Castrejón-Téllez

**Affiliations:** 1 Department of Cardiovascular Biomedicine, Instituto Nacional de Cardiología Ignacio Chávez, Juan Badiano 1, Sección XVI, Tlalpan, México City 14080, Mexico; 2 Department of Immunology and Research Direction, Instituto Nacional de Cardiología Ignacio Chávez, Juan Badiano 1, Sección XVI, Tlalpan, México City 14080, Mexico; 3 Department of Reproductive Biology, Instituto Nacional de Ciencias Médicas y Nutrición Salvador Zubirán, Vasco de Quiroga 15, Sección XVI, Tlalpan, México City 14000, Mexico; 4 Department de Instrumentation Electromechanically, Instituto Nacional de Cardiología Ignacio Chávez, Tlalpan, México City 14080, Mexico; 5 Department of Pharmacology, Instituto Nacional de Cardiología Ignacio Chávez, Juan Badiano 1, Sección XVI, Tlalpan, México City 14080, Mexico; 6 Department of Physiology, Instituto Nacional de Cardiología Ignacio Chávez, Juan Badiano 1, Sección XVI, Tlalpan, México City 14080, Mexico

**Keywords:** *Hibiscus sabdariffa* L., arachidonic acid, cyclooxygenase metabolism, metabolic syndrome, kidney

## Abstract

The renal system is engaged in metabolic syndrome (MS) and metabolites of arachidonic acid (AA) participate in renal homeostasis and disruption of functionality. *Hibiscus sabdariffa* L (HSL) is used as a diuretic and could improve renal function. The aim of this study was to assess if treatment with HSL at 2% improves renal function in MS through the metabolites of AA. A total of 24 male Wistar rats were divided into four groups: Group 1, control (C); Group 2, MS with 30% sucrose in drinking water, Group 3, MS plus HSL infusion at 2% (MS+HSL); and Group 4, C+HSL. We evaluated the perfusion pressure changes (∆-PP), the activities of cyclooxygenases (COXs), the percentage of AA, the expressions of PLA_2_, COX2, COX1, 5-LOX, TAXS and CYP450, and the concentrations of prostaglandins in the kidney from rats with MS. There was a decrease in the ∆-PP, in the activities of COXs, and the expressions of COX2 and CYP450 (*p* ≤ 0.03, respectively)as well asPGE_2_, TxB_2_, and LKB_4_ (*p* ≤ 0.01, respectively). However, the percentage of AA and expressions of PLA_2_ and PGE1 (*p* = 0.01, respectively) were increased in C and MS+HSL. The HSL treatment improved the function and anatomical structure of the kidneys in the MS rats, through antioxidant molecules, and inhibited the pathways that metabolize the AA including that of PLA_2_, COX2, 5-LOX, TAXS, and CYP450 while favoring the COX1 pathway. This improves the vascular resistance of renal arterioles.

## 1. Introduction

Metabolic syndrome (MS) is a cluster of three or more pathologies that include hypertension, dyslipidemia, obesity, overweight status, insulin resistance, hyperinsulinemia, and type2 diabetes. It is largely influenced by environmental factors such as an inadequate diet, rich in carbohydrates and/or saturated fat, and the lack of physical activity [1]. The coexistence of these pathologies in the same patient causes a long-term inflammatory state, and an oxidative background with deterioration of organs and systems, including the renal system [1].The kidney maintains a stable extracellular environment that supports the function of all cells in the body; it controls the water and ionic balance, and the excretion of sodium, chloride, potassium, calcium, magnesium, and phosphate. It also manages the acid–base status and regulates the excretion of other substances such as serum creatinine. The fundamental unit of the kidney is the nephron in which the glomerulus acts as the filtering unit or sieve, keeping proteins and cells in the bloodstream but allowing the extra fluid and wastes to pass through it.

Four enzymatic pathways are involved in the metabolism of arachidonic acid (AA) in the kidney [2]. These pathways are mediated by the cyclooxygenase isoforms (COX1 and 2), the 5-, 12-, and 15-lipoxygenase (LOX) isoforms, and cytochrome p450 (1 to 4) (CYP450). Each enzyme has several subtypes and isoforms. AA is metabolized to prostaglandins E_2_ (PGE_2_) with vasoconstrictor and inflammatory effects by the COX2 pathway, and to prostacyclin I_2_ (PGI_2_) with a vasodilator effect by the COX1 pathway [3]. AA is converted to hydroperoxyeicosatetraenoic acids (HPETES) that are then reduced to the hydroxyeicosatetraenoic acids (HETEs) and leukotrines (LTs as LTB_4_, LTC_4_, and LTE_4_) by the LOX isoforms. These last enzymes are related to an increased entry of Ca^2+^ into cells [4]. The CYP450 family produces cis-epoxyeicosatrienoic acid (EETs) with vasoconstrictor effects [5]. In addition, AA can give rise to 8-isoprostanes, which are pro-inflammatory in a pro-oxidant environment [6]. Finally, thromboxane synthase (TXAS) gives rise to thromboxanes (TxB), with a pro-aggregation and thrombotic effect. However, AA must first be released from the phospholipids (PL) of the cell membrane with the action of the family of phospholipases (PLA_2_) before entering into these routes [7].

On the other hand, natural products can improve human health. As an example, Chlorella algae, a blue-green microalgae, may restore the function of pancreatic insulin-secreting cells in diabetic rats treated with streptozotocin [8]. Also, dandelion flowers have antioxidant properties that prevent chronic inflammation and development of obesity, cancer, and numerous cardiovascular risk factors [9]. Saponins, a diverse class of natural compounds present in some plant species, have the ability to inhibit various cancers in vitro and in vivo [10]. A supplement based on lipids and enriched with micronutrients improves the birth weight, gestational age, and length and head circumference of new-born babies in pre-eclamptic women [11].In this sense, *Hibiscus sabdariffa* L. (HSL) has been used as a medicinal herb in Africa, Asia, and Latin America against hypertension, urinary blander, and kidney stones, as a diuretic and against liver disorders among other pathologies. HSL calyxes contain many chemicals with antioxidant properties that include polyphenols, flavonoids, and anthocyanins [12]. The beneficial proprieties of HSL in the kidney, as a crude extract or as an infusion, have been attributed to the vasorelaxant effect of anthocyanins probably with the inhibition of the Ca^2+^ channels and to its action on the angiotensin converting enzyme (ACE). In this sense, treatment with the HSL extract reduced the activity of ACE in plasma and decreased the concentrations of sodium without modifying potassium levels in hypertensive patients [13]. Polyphenols extracted from HSL inhibit the inflammation induced by lipopolysaccharides in vitro in macrophages by decreasing the expression of COX2 [14]. Another study demonstrated that the catechin of HSL reduced albuminuria and normalized interstitial fibrosis in the kidney in diabetic rats treated with streptozotocin [13]. Our group reported that the HSL infusion at 2% decreases oxidative stress (OS), increases the total antioxidant capacity, decreases albuminuria, and protects renal function in an MS rat model [15].

Our model of MS in Wistar rats is the result of chronic consumption of 30% commercial sucrose in drinking water for 20–24 weeks. This model courses with hypertension, hypertriglyceridemia, central obesity, OS, hyperinsulinemia, insulin resistance (IR), renal dysfunction, and damage to other organs [16]. We also reported that the metabolism of AA is altered in this model, having an increased expression/activity of COX2 and a decreased expression/activity of COX1 associated with a reduction in the total AA as a free form and in the PL [17]. However, to date, there exists no studies on the effects of HSL on the metabolic pathways that use AA and their possible contribution to improve renal function in MS, despite the findings reported in the literature. Therefore, the aim of this study was to try to understand and elucidate how treatment with HSL at 2% contributes to improve the renal function through the pathways that metabolize AA in a rat model with MS.

## 2. Results

### 2.1. Determinations of Total Flavonoids, Anthocyanins, Polyphenols, and Vitamin C

The determinations of total flavonoids, anthocyanins, polyphenols, and vitamin C in the HSL infusion were made with the techniques reported by Jagota, Jia, Lee’s, and Sánchez-Rangel, respectively. The HSL infusion at 2% contained cyaniding-3-glucoside (142.06 ± 25.9 mg/L), quercetin (0.38 ± 0.02 mg/L), polyphenols (0.50 ± 0.03 mM/L), and vitamin C (0.53 ± 0.02 mg/L). In addition, the rats consumed cyanidin-3-glucoside, quercetin, and polyphenols at approximately 141.06 ± 25.97, 0.38 ± 0.03, and 0.50 ± 0.01 mg/day, respectively.

### 2.2. Biochemical Markers in Serum

The MS rats showed significant increases in insulin, the HOMA index, TG, intra-abdominal fat, and SBP vs. control rats (*p* = 0.001). The treatment with HSL infusion at 2% in MS rats significantly decreased insulin, the HOMA index, TG, and SBP (*p* ≤ 0.04) in comparison with the MS rats. In the C group, the HSL treatment did not show significant differences in any of the biochemical parameters determined. The glucose levels did not show significant changes in any of the groups (Table 1).

### 2.3. Markers of the Renal Function

Table 2 represents the indicators of renal function in the experimental groups, where the MS rats showed deterioration of the renal function in comparison with the C and MS+HSL groups. This was evidenced by a significant increase in CCr, albuminuria, and urine volume of 24 h (*p* ≤ 0.001). In addition, the MS rats showed an increase in water consumption in comparison with the C and MS+HSL groups (*p* ≤ 0.001 and *p* = 0.01, respectively). However, the treatment with HSL infusion at 2% did not produce changes in this parameter in the C+HSL group. The weight of the right kidney in the groups did not show a significant difference.

### 2.4. Prostaglandins

Table 3 shows the prostaglandin values in the experimental groups where significant increases in PGE_2_ and TxB_2_ were present in the MS rats in comparison with C and MS+HSL rats (*p* = 0.001 and *p* = 0.01, respectively). A tendency to increase was observed in LKB_4_ in MS rats in comparison with C rats (*p* = 0.09) but there was a decrease (*p* = 0.01) in comparison with the MS+HSL rats. In the same sense, the 6-keto-PGF_1α_ was decreased in the MS rats in comparison with C and MS+HSL rats (*p* = 0.01). The same tendency was observed in the AA percentage between MS vs. C and MS+HSL rats (*p* = 0.001 and *p* = 0.01, respectively). The HSL treatment did not modify the prostaglandin levels, or the AA percentage in the C+HSL group.

### 2.5. Delta of the Perfusion Pression

**Figure 1:**
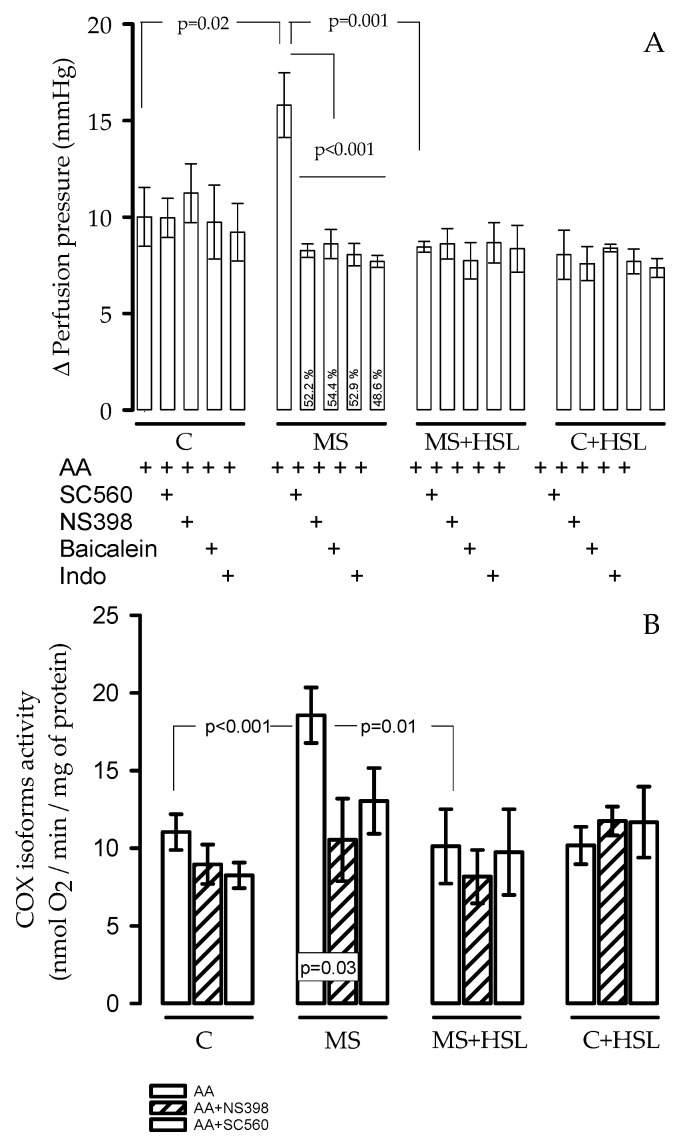
Panel (**A**) shows the changes in the Δ of perfusion pression (Δ-PP), by infusing 4 µg of AA, which is the substrate for the enzymes that metabolize it to eicosanoids with vasoconstrictive, prothrombotic, aggregating, or vasodilatory effects in the kidney of the rat experimental groups. The Δ-PP was significantly increased in MS rats in comparison with C and HSL rats. Panel (**B**) shows the activity of the COX isoforms in the presence of 100 µM of AA alone or with the use of the specific inhibitors, in the microsomal fraction of the kidney homogenate. The use of inhibitors in this fraction allows for discriminating between the activities of the two COX isoforms involved in the metabolism of AA. Values are expressed as mean ± SE (*n* = 6). Abbreviations: C = control, HSL = *Hibiscus sabdariffa* L., MS = metabolic syndrome.

Figure 1A shows the changes in the Δ-PP, in the experimental groups. The Δ-PP was significantly increased in MS rats in comparison with C rats (10.0 ± 1.5 mmHg vs. 15.7 ± 1.6 mmHg, *p* = 0.02). The HSL treatment in MS rats resulted in a significant decrease in the Δ-PP compared to MS rats (8.4 ± 0.2 mmHg vs. 15.7 ± 1.6, *p* = 0.001). The Δ-PP in the kidney of the MS rats when the AA was perfused in the presence of NS398, SC560, baicalein, and Indo decreased by 52.2%, 54.4%, 52.9%, and 48.6%, respectively, showing a significant difference (*p* ≤ 0.001) in comparison to the condition without the inhibitors. The perfusion of the AA in the presence of the inhibitors did not show differences in the percentage of the Δ-PP in C, MS+HSL, and C+HSL rats.

### 2.6. Coxs’activities

Figure 1B shows the activity of the COX isoforms in the microsomal fraction of the kidney homogenate in each of the experimental groups. The use of the specific inhibitors in this fraction allowed fordiscriminating between the activities of the two COX isoforms involved in the metabolism of AA. The results showed a significant increase in the MS rats when compared to the C and the MS+HSL groups (*p* < 0.001, *p* = 0.01, respectively). In the presence of NS398, which is a specific inhibitor of COX2, there was a decrease in its activity in the MS group in comparison with the same group without the inhibitor (*p* = 0.03). However, the other experimental groups did not show statistical changes. In the presence of SC560, which is a specific inhibitor of COX1, the activity of the enzyme did not show statistical changes in the MS group in comparison with the same group without the inhibitor. In the other experimental groups, the presence of the inhibitors did not show statistical changes.

### 2.7. Enzymatic Expressions

**Figure 2:**
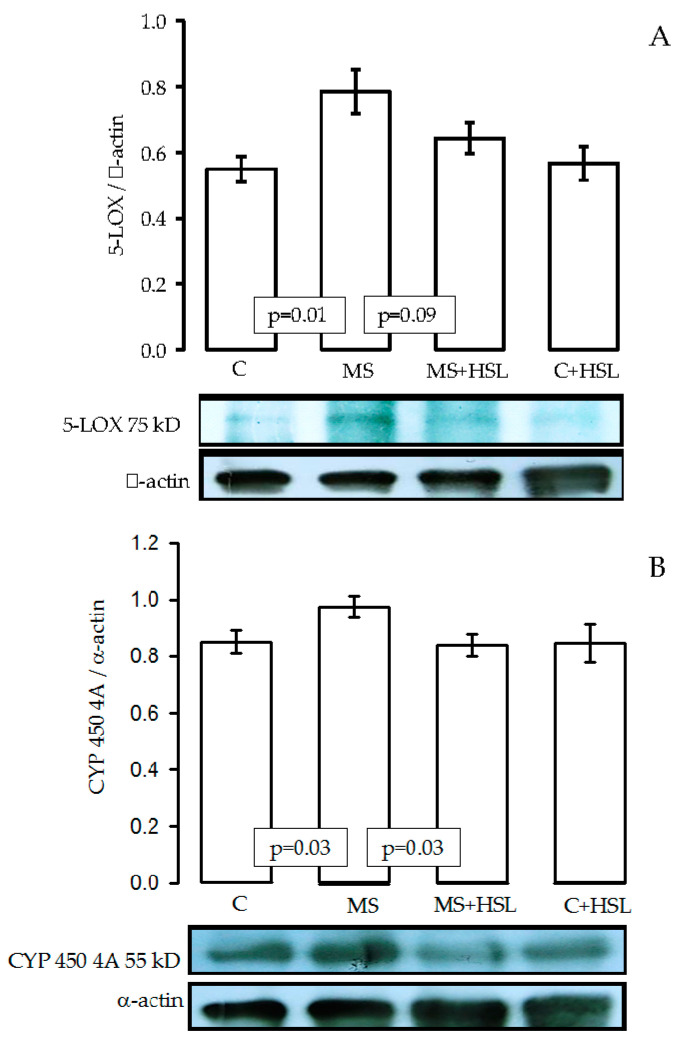
Panel (**A**,**B**) show the expressions of 5-LOX and CYP450 4A, respectively, in the kidney homogenates in the MS group in comparison with the C and HSL groups. Values are expressed as mean ± SE (*n* = 6). Abbreviations: C = control, HSL= *Hibiscus sabdariffa* L., MS = metabolic syndrome.

**Figure 3:**
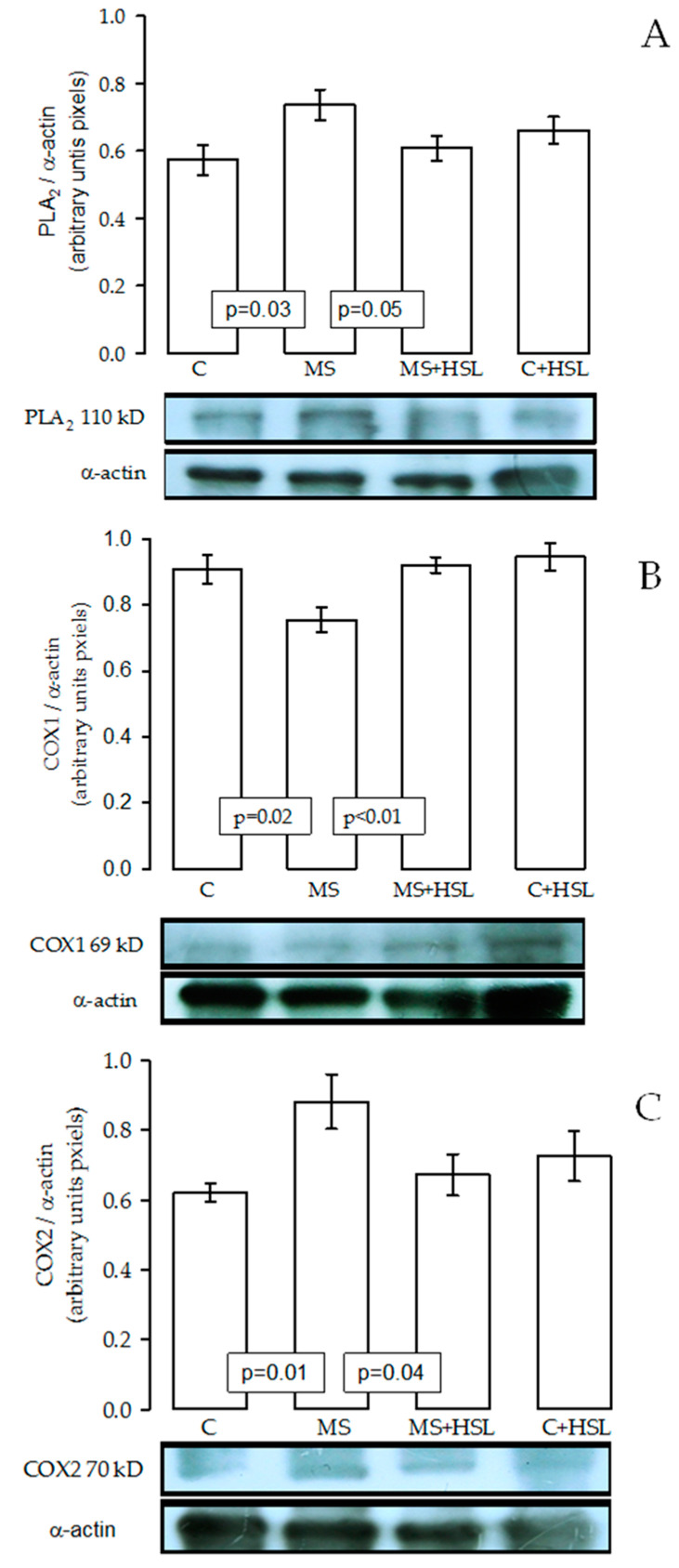
Panel (**A**–**C**) show the expressions of PLA_2_, COX1, and COX2, respectively, in the kidney homogenates in the MS group in comparison with the C and HSL groups. Values are expressed as mean ± SE (*n* = 6). Abbreviations: C = control, HSL = *Hibiscus Sabdariffa* L., MS = metabolic syndrome.

Figure 2A,B show an increase in the expression of 5-LOX (*p* = 0.01) and CYP450 4A (*p* = 0.03) in the kidney homogenates from the MS group in comparison with the C group. The treatment with HSL at 2% showed a tendency to decrease the expression of 5-LOX (*p* = 0.09) in MS rats. There was a statistical change in CYP450 4A (*p* = 0.03). Figure 3A,B show an increase in the expressions of PLA_2_ (*p* = 0.03) and COX2 (*p* = 0.01), but a decrease in COX1 (*p* = 0.02, Figure 3C) in the MS group in comparison with the C group. The treatment with HSL at 2% showed a tendency to decrease the PLA_2_ and COX2 expressions (*p* = 0.05) in MS rats, but there was an increase in the COX1 expression (*p* = 0.04).

### 2.8. Histological Changes

**Figure 4:**
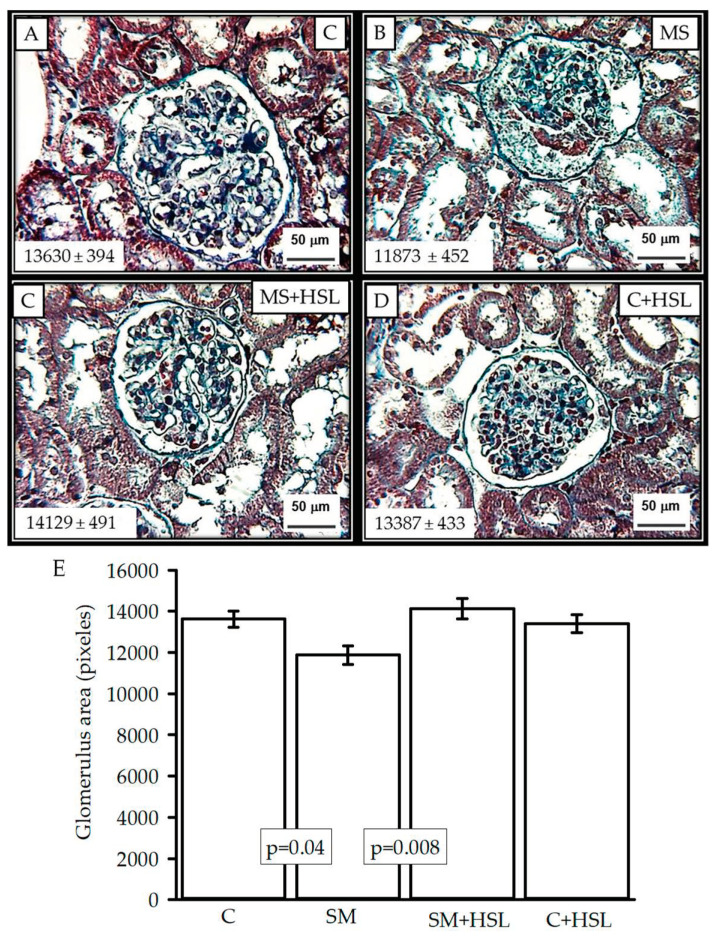
Representative photomicrographs of the rat renal cortex. Images show the glomerulus from Panel (**A**) C rats, Panel (**B**) MS rats, Panel (**C**) MS+HSL rats, and Panel (**D**) C+HSL rats. No abnormalities were observed under light microscopy in C, MS+HSL, and C+HSL groups, where glomerular spaces and loops with their fine and delicate membrane are preserved. However, in the glomerulus from the MS rats, there was fibrosis, retracted glomerular tufts, and increased urinary spaces with detritus. Masson’s trichrome stain at 400×. Panel (**E**) shows the densitometrical analysis where a significant decrease in the size of the glomerulus in the MS rats is observed when compared to the C and the MS+HSL groups.

Figure 4A–D show representative histological sections of a glomerulus in the C, MS, MS+HSL, and C+HSL groups, with the average area size of the glomerular tang expressed in arbitrary units (pixels). The densitometrical analysis showed a significant decrease in the size of the glomerulus in the MS rats when compared to the C and the MS+HSL groups (*p* ≤ 0.03, panel E). The glomerular spaces and loops with their fine and delicate membrane are preserved in the C, MS+HSL, and C+HSL groups in comparison with those from MS rats, which show fibrosis, retracted glomerular tufts, and increased urinary spaces with detritus.

## 3. Discussion

The renal system shows different alterations in MS that result in proteinuria, infiltration of inflammatory cells, accumulation of lipids, sclerosis, and a decrease in the CCr. The production of eicosanoids through the metabolism of AA is important in the renal system, since it participates in the maintenance of the proper relaxation and contraction of the renal vasculature in the glomeruli, thin limb of the loop of Henle, connecting tubule, collecting ducts, and mesangial cells [17,18]. The traditional medicinal herb, HSL, has been used to treat different pathologies of the renal system such as urinary bladder and kidney stones, and as a diuretic [19]. Its beneficial effects have been attributed mainly to the antioxidants it contains and to its activity on the ACE. However, only a few investigations have addressed the effect of HSL on enzymes that synthesize eicosanoids including CYP450 isoforms or COX2 [20]. For this reason, the aim of this study consisted of studying how the treatment with HSL at 2% contributes to improve renal function through the pathways that metabolize AA in a rat model of MS.

AA, which is a substrate for the enzymes studied in this paper, has to be released from the PL of the cell membrane by PLA_2_ before it is metabolized. This enzyme is Ca^2+^-dependent. Our results show that the treatment with HSL resulted in a decrease in the expression of PLA_2_, probably through the antioxidants it contains that are capable of modulating intracellular Ca^2+^ concentrations via the inhibition of calcium channels. As a result of the decreased expression of this enzyme, the percentage of AA in PL is increased. Another possible explanation for the increase in the AA percentage in MS rats treated with HSL is the fact that HSL contains linoleic and oleic acids [21], which are transition fatty acids that may be desaturated by the 5 and 6 desaturases, resulting in the formation of AA, which may then be reinserted in the cell membrane to increase fluidity [16]. Our MS rat model also courses with a decrease in the percentage of AA in total fatty acids and in those in the PL [17].

Our MS model in rats also has alterations in the activities or expressions of the COX isoforms and TXAS in the kidney [17]. In this sense, the treatments with HSL at 2% result in a decrease in the activity/expression of COX2 and an increase in the activity/expressions of COX1 in MS rats. This leads to a decrease in PGE_2_ and an increase in PGE_1_. These prostaglandins modulate the vascular tone in the afferent and efferent arterioles of the glomerulus, leading to an increase in CCr, and a decrease in albuminuria. Therefore, glomerular filtration is improved, as shown by our results. It has been previously reported that the polyphenols from HSL may reduce the expression of COX2 in LPS-treated macrophages.This enzyme also contributes to a pro-inflammatory state in hypertension. Our results confirmed these results and also showed that the treatment with HSL can favor the increase in the COX1 activity/expression. COX1 increases the synthesis of PGE1 (6-keto-PGF_1α_ metabolite stable of PGE1) with vasodilator, antithrombotic, and anti-inflammatory effects and contributes to the vasodilation of arterioles in the glomerulus that lowers systemic SBP [22].

In addition, there was a decreased expression of 5-LOX and CYP450 4A with the treatment with HSL at 2% in the homogenate of the kidney from MS rats. The eicosanoids produced by these enzymatic pathways such as LKB_4_ are mediators of inflammation and contraction in the mesangial cells, the limb of the loop of Henle, and the collecting ducts. An increase in this prostaglandin can reduce the glomerular ultrafiltration coefficient [23]. Therefore, our results suggest that the HSL treatment may modulate the expression of these enzymes, which results in a decrease in its metabolites as previously reported [5]. In this sense, the ethanolic HSL extract causes inhibition of some CYP isoforms in vitroin human liver microsomes [24]. Another study showed that this effect may be through the protocatechuic acid and phenolic acids that are present in high concentrations in HSL [25]. Likewise, polyphenols such as quercetin, lignin, lignan, kaempferol, resveratrol, and catechins, among others, are capable of acting on the same metabolic pathways [22], and have the capacity to inhibit other enzymes of the AA metabolism such as PLA_2_, 5-LOX, TXAS, and COX2 [26].

Furthermore, our results suggest that TxB_2_, which is a metabolite of AA produced by TXAS, was decreased with the HSL treatment. The participation of this metabolite alongside with LKB_4_ and PGE_2_ may induce the transcription of laminin, fibronectin, and IV collagen, in the glomerular basement membrane with a loss of the electrical charge, and therefore result in a pro-inflammatory state, albuminuria, and sclerosis in the kidney [27,28].

The expressions of COX2 and COX1 that were modified in the kidney homogenate were reflected in the activities of the COX isoforms when using specific inhibitors for both COX2 and COX1 (NS398 and SC560, respectively). In the presence of AA, the microsomes of the kidneys from rats with MS showed that the oxygen consumption was increased, but the oxygen consumption decreased (the molecular O_2_ is incorporated to AA to synthetize prostaglandins) in the presence of NS398. This suggests and supports our results of the overexpression of COX2, which participates in a larger proportion than COX1. This enzyme was not inhibited in a similar manner to COX2. However, the treatment with HSL in rats with MS did not reflect this change. This result suggests that the activities of these enzymes can be modulated using the components contained in the HSL infusion such as polyphenols, flavonoids, anthocyanins, and vitamin C, among others, resulting mainly in a decrease in COX2 activity and favoring COX1 activity.

The increase in the expression and activity of the different enzymes present in the kidney may be evaluated on the dynamics of renal function in the isolated and perfused kidney. This system evaluates renal vascular resistance in response to different drugs and metabolites resulting in changes in the ∆-PP that are reflected in glomerular filtration [28]. The results obtained here show that there is a larger vascular resistance in the kidney from the MS rats associated with the alteration in the enzymatic pathway that metabolizes AA. These changes lead to damage in the renal function. This isaccompanied by a loss of glomerular filtration rate and an increased albuminuria [5,16,17]. This was evidenced with the inhibition of the percentage of the different metabolic pathways (COX2 (52.2%), COX1 (54.4%), CYP450 (52.9%), and Indo (48.6%), with an unspecific inhibitor). However, the HSL treatment showed no significant changes in the inhibition percentage in the kidney of the rats from the MS model, but decreased the vascular resistance (reduced ∆-PP) in them. This suggests that the antioxidants present in the HSL infusion modulate the activities of the enzymes that metabolize the AA, and as a consequence, they modulate the production of the vasoconstrictor and vasodilatation eicosanoids. As an example, baicalein is a flavonoid that inhibits CYP450 [29]. Both the decrease in the vasoconstrictors and the increase in vasodilators resulting from these pathways act through the endothelium of the renal arterioles and modify the vascular tone, favoring vasodilation. Therefore, they improve the glomerular filtration rate. Our results show that the prostaglandins that are decreased using the HSL treatment and that can have an effect are PGE_2_, LKB_4_, and TxB_2_; however, PGE_1_ was increased.

The alterations of the dynamics of renal function are also accompanied by structural and anatomical modifications, which are evidenced with the histological changes in the glomeruli from MS rats, as previously reported [5,16,17]. However, treatment with HSL in MS rats contributed to the reduction in these modifications.

Last but not least, the MS rat model courses with hypertriglyceridemia, intra-abdominal fat, IR, hyperinsulinemia, and hypertension as previously reported [5,16,17]. However, the treatment with the HSL infusion decreased intra-abdominal fat, hypertriglyceridemia, hyperinsulinemia, and IR in the MS rats. These changes can be attributed to the flavonoids and polyphenols, present in the HSL infusion. These components can block lipid accumulation in the adipose tissue and the liver [30]. They may also increase the β-oxidation of fatty acids by inhibiting fatty acid synthase, cholesterol acyltransferase, 3-hydroxy-3-methylglutaryl coenzyme A reductase, and acyl-coenzyme A. These changes result in a decrease in insulin sensitivity and reduce the triglyceride levels and intra-abdominal fat [31]. This can contribute to decrease the structural and physiological abnormalities in the kidney, such as the increased tubulointerstitial damage, fibrosis, and mesangial proliferation [32,33].

**Figure 5:**
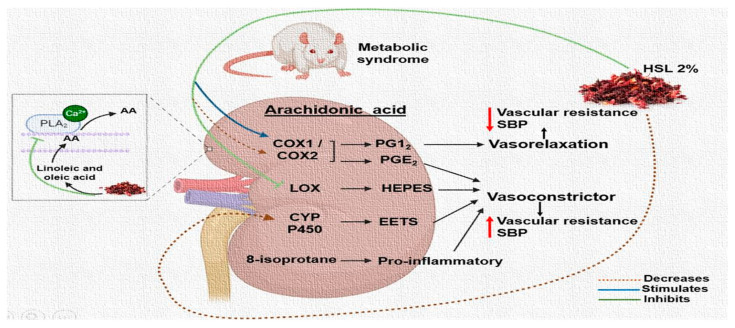
Summary of the possible mechanisms by which the HSL infusion at 2% acts on the enzymatic pathways that employ the AA in the kidney in an MS rat model. Uparrow = increase, down arrow = decrease.

Figure 5 shows the possible mechanisms by which HSL infusion at 2% may down-regulate the metabolism of AA in the kidney of the rats with MS.

## 4. Materials and Methods

The study was designed and carried out in compliance with the Laboratory Animal Care Committee of the National Institute of Cardiology Ignacio Chávez in México (INC/CICULA/011/2019) for the experiments in animals. Experiments were conducted in compliance with the Guide for the Care and use of laboratory animals of the National Institutes of Health (NIH). A total of 24 male Wistar rats were used to form 4 groups with 6 animals each for 20 weeks, and each group was treated as follows: Group 1: control (C, tap water); Group 2: MS with 30% sucrose in their drinking water, Group 3: MS with 30% sucrose plus HSL infusion (MS+HSL) at the concentration of 20 g/L (2%), and Group 4: C plus HSL at 2%. The animals were housed in ad hoc plastic boxes (Nalgene, New York, NY, USA). The animals were kept for 4 weeks under the following conditions: 12 h light/12 h dark cycle, environment temperature, and relative humidity between the ranges of 18–26 °C and 40–70%, respectively. Commercial rodent feed that contained 6% crude fiber, 4.5% crude fat, 23% crude protein, 2.5% minerals, and 8% ash (Labdiet 5008; PMI Nutrition International, Richmond, IN, USA) was provided freely. The HSL infusion at different percentages was administered orally (ad libitum) in drinking water.

### 4.1. Systolic Blood Pressure (SBP)

The SBP was determined at the end of the experimental period in the rats and it was measured using a tail cuff attached to a pneumatic pulse transducer method (Narco Bio-Systems Inc., Houston, TX, USA) [17]. Briefly, the rats were immobilized in an acrylic device that allowed them to breathe freely, and were rested on a plate that kept them warm. This device left the tail free to apply the tailcuff. The animals were left to relax for 15–20 min and five measurements of SBP were carried out on each rat.

### 4.2. Preparation of the HSL Infusion

The HSL calyces were acquired in Chilapa de Alvarez (Guerrero, México). In total, 20 g of the HSL calyces was added to a liter of boiling (90–100 °C) drinking water for 10 min and then left to cool; subsequently, 300 g of sucrose was added. The solution was filtered and stored at 4 °C for consumption by the rats of Group 3. The HSL infusion at 2% for Group 4 had no sucrose added [16,17,34].

### 4.3. Determinations of Polyphenols, Total Flavonoids, Total Anthocyanins, and Vitamin C in HSL Infusion

The determination of total flavonoids was conducted with the Jia method [35]. The calibration curve was made using quercetin as a standard and the absorbance was read at 510 nm. The polyphenol determination was made with aFolin–Ciocalteu reagent [36]. Determination of total anthocyanins was performed with Lee’s method. The absorbance was measured at 520 nm and 700 nm and compared with a blank cell containing distilled water. The absorbance difference was performed to calculate cyaniding-3-glucoside [37]. Estimation of vitamin Cwas through the Folin–Ciocalteu reagent at 0.20 mM. The absorbance was measured at 760 nm [38].

### 4.4. Biochemical Variables

Commercial kits were used for the determination of some serum biochemical variables in the rats. Glucose concentration was determined with an enzymatic SERA-PAKR Plus kit (Bayer Corporation, S’ees, France). Total cholesterol (TC) and triglycerides (TG) determinations were made using commercial enzymatic kits (RANDOX Laboratories Ltd., Crumlin, County Antrim, UK). Insulin was determined using commercial radioimmunoassay kits (RIA) (Linco Research Inc., Saint Charles, MO, USA). The HOMA index of IR was calculated. HOMA−IR = insulin μU/mL × glucose mM/L/22.5.

### 4.5. Albuminuria and Creatinine Depuration

Albuminuria was measured using the bromocresol green reagent [39]. Serum creatinine (UCr and SCr, respectively) was measured with the Jaffe method [40] and glomerular filtration was calculated according to the following formula: clearance creatinine (CCr) = [urine creatinine]/[serum creatinine] × urinary volume (24 h)/time (1440 min).

### 4.6. Isolated Perfused Kidney

The rats were anesthetized with an intraperitoneal injection of sodium pentobarbital (63 mg/kg body weight). The right kidney was exposed using midline laparotomy, and the mesenteric and right renal arteries were cleared of surrounding tissue. The right renal artery was cannulated through the mesenteric artery to avoid interruption of blood flow, and the kidney was removed, suspended, and perfused at constant flow by means of a peristaltic pump (MasterFlex Easy-load II, no. 77200-50; Cole-Parmer Instrument Co, Vemon Hills, IL, USA) with a Krebs solution (in millimoles per liter)—118 of NaCl, 1.2 of NaH_2_PO_4_, 25 of NaHCO_3_, 4.7 of KCl, 1.2 of CaCl_2_, 4.2 of MgSO_4_, and 5.5 of glucose (pH 7.4)—with a constant flow at 37 °C and oxygenated with 95% O_2_/5% CO_2_. The basal perfusion pressure (PP) was adjusted to 80–90 mmHg. The mean flow rate of the perfusing solution was 8 to 9 mL/min. The PP was measured with a transducer (Grass Telefactor, Grass Technologies, Astro Med, West Warwick, RI), captured, and recorded by means of a Grass model polygraph 79D and an online program (Grass PolyView). Data are expressed as changes (Δ) of PP in millimeters of mercury (mmHg). After at least 15 min of perfusion and once a stable PP had been obtained, vasoconstrictor responses to 4 μg/(mL min) of AA (Sigma Aldrich Darmstadt, Alemania) were determined in the absence and presence of 10 μmol/L of indomethacin (Indo, unspecific inhibitor of the COX pathway), 10 μmol/L of NS398 (COX2 selective inhibitor), 10 μmol/L of SC560 (COX1 selective inhibitor), or 100 μmol/L of baicalein (pathway inhibitor of CYP450). These doses were selected from published data as they seemed the most convenient after dose–response curves were obtained. Changes in the PP produced by the AA at 4 µg and the inhibitors were calculated by taking the mean of the pulsatile traces before administration and the mean of the traces at the maximal PP value after administration. Data are expressed as delta changes (Δ) of PP in mmHg. After each perfusion bolus, the kidneys were washed for a period of 20 min with a Krebs solution, to allow the organ to return to the basal PP (80–90 mmHg), and no sign of tachyphylaxis was present [16,17,41].

### 4.7. Kidney Homogenate

The left kidney was dissected and washed with a 0.9% saline solution. The capsule was removed, and cut in half. A half of the kidney was homogenized in a sucrose buffer (25 mM of sucrose, 10 mM of Tris, 1 mM of EDTA, and pH 7.35) with protease inhibitors (1 mM of PMSF, 2 μM of pepstatin, 2 μM of leupeptin, and 0.1% aprotinine). The homogenate was kept in ice. The kidney homogenate was centrifuged at 900× *g* for 10 min at 4 °C. The supernatant was separated and stored at −30 °C until required. Total proteins were determined with the Bradford method [42].

### 4.8. Microsomes

In total, 500µg of the protein of the kidney homogenate was used and suspended in 500 μL of 0.1 mol/L and centrifuged at 8000× *g* for 10 min at 4 °C. The pellet was discarded, and the supernatant was centrifuged at 44,000× *g* for 1 h at 4 °C; the resultant pellet was suspended in 250 μL of a 0.1 mol/L phosphate buffer at pH 7.35. The microsomes were used to determine the activities of the COX isoforms [17].

### 4.9. Cyclooxygenase Activity Assays

COXs’ activities were studied by monitoring the rate of O_2_ uptake using an oxymeter (YSI oxymeter model 5300A-1), which was coupled to a Clark-type electrode. To initiate the reaction, 3 mL of a 0.1 M Tris-HCl buffer, 1 mM of phenol, 85 μg of bovine hemoglobin (pH 8), and 100 μM of AA at 37 °C were added to 50 μg of kidney microsomes. Inhibition and discrimination of the catalytic activity of COX2 and 1 was performed with the addition of 10 μM of NS398, and SC560, respectively. The calibration curve was obtained with human COX2 provided by Sigma-Aldrich. A unit of cyclooxygenase activity is defined as the ability of the enzyme to catalyze oxygenation of 1 nmol of AA per minute at 37 °C.

### 4.10. Prostaglandins

Prostaglandins were extracted from the kidney homogenate by adding 0.5 mL of water–ethanol (1:4 vol/vol) and 10 μL of glacial acetic acid to 25 µg of protein. The mixture was well shaken and incubated at room temperature for 5 min and centrifuged at 2500× *g* for 5 min. The supernatant was applied to a Sep-Pak C18 mini-column (Millipore, Billerica, MA, USA) previously equilibrated with 2 mL of 10% ethanol. The column was then washed with 1 mL of water followed by 1 mL of hexane, and prostaglandins were eluted with 1.50 mL of ethyl acetate. The samples were dried under a nitrogen stream and re-suspended in 300 μL of phosphate-buffered saline–ethanol (2:1 *vol/vol*) [14]. The prostaglandins were determined with ELISA kits; prostaglandin E (Item No. 514531), 6-keto prostaglandin F_1α_ (Item No. 515211), leukotriene B_4_ (Item No. 520111), and thromboxane B_2_ (Item No. 501020) were obtained from Cayman Chemical (Ann Arbor, MI, USA), and were read in a visible light micro plate reader at 492/630 nm.

### 4.11. Extraction and Derivatization of the AA of the Phospholipids

For the extraction and derivatization of the AA of the phospholipids, 50 μg of L-α-phosphatidylcholine-di-heptadecanoyl acid as an internal standard and 1 mL of acetone were used. The mixture was shaken vigorously in a vortex for 30 s and centrifuged at 1145× *g* at room temperature for 4 min. The supernatant was removed, and the button was suspended with chloroform–methanol (2:1, *vol/vol*) with 0.002% BHT, according to the method described by Folch [43]. The AA was trans-esterified to its AA methyl esters by heating them at 90 °C for 2 h with 2 mL of methanol plus 0.002% BHT, 40 μL of H_2_SO_4_, and 100 μL of toluene. They were separated and identified with gas chromatography-FID in a Carlo Erba Fratovap 2300 chromatograph equipped with a capillary column packed with the stationary phase, HP-FFAP (description: 30 m length × 0.320 mm diameter × 0.25 μm film), and fitted with a flame ionization detector at 210 °C, with helium as the carrier gas at a flow rate of 1.2 mL/min. The areas under the peaks were calculated with a Shimadzu C-R6A Chromatopac integrator coupled to the gas chromatograph.

### 4.12. Western Blotting for COXs’Isoforms, CYP4A, 5-LOX, and PLA_2_

An amount of 25 μg of protein of the kidney homogenate was run on 12% SDS-PAGE, blotted onto a polyvinylidene difluoride membrane (0.22 μm, Millipore, Billerica, MA, USA), and then blocked for 1 h at room temperature with Tris-buffer-solution–0.01%-Tween (TBS-T 0.01%) and 5% non-fat milk. The membranes were incubated overnight at 4 °C with mouse primary monoclonal antibodies: a COX2 antibody (H-3: sc-376861), COX1 antibody (H-1: sc-166573), and CYP4A1/A2/A3 antibody (clo4: sc-53247), providedfrom Santa Cruz Biotechnology, Santa Cruz, CA, USA;an Anti-5 Lipoxygenase/5-LO antibody (EP6072(2), ab169755) provided from abcam; and C-PLA2 (#SAB4502200) provided from Sigma Aldrich at a dilution of 1:5000, respectively. Then, the membranes were incubated overnight at 4 °C with a secondary antibody conjugated with horseradish peroxidase at a dilution of 1:10,000 (Santa Cruz Biotechnology, Santa Cruz, CA, USA). All of the blots were incubated with an α-actin antibody as a load control. The enzymes were detected with a chemiluminescence assay (Clarity Western ECL Substrate, Bio-Rad Laboratories, Inc., Hercules, CA, USA). Chemiluminescence that was emitted in this process was detected in X-ray films (AGFA, Ortho CP-GU, Agfa HealthCare NV, Mortsel-Belgium). Images from each film were acquired with a GS-800 densitometer (including Quantity One software from Bio-Rad Laboratories, Inc., Hercules, CA, USA). The values of the density of each band are expressed as arbitrary units (AU).

### 4.13. Histopathological Analysis

Tissue was processed for light microscopy according to standard techniques. A segment of the kidney was dissected, decapsulated, and washed in 0.9% NaCl for 30 s, fixed in a 10% formalin solution for 24 h, gradually dehydrated in ethanol, cleared in xylene, and embedded in paraffin. The kidney was cut into five-micrometer-thick slices with a microtome (Leica RM212RT, Wetzlar, Germany); the paraffin sections were stained with Masson’s trichrome. Histological sections were analyzed at 400× magnification using a model 63,300 light microscope (Carl Zeiss, Oberkochen, Germany), equipped with a Tucsen (9 megapixels) digital camera coupled to TSview 7.3.1 software. The glomerular area was analyzed with densitometry using Sigma Scan Pro 5 Image Analysis software (Systat Software Inc., San Jose, CA, USA). The density values are expressed as pixel units.

### 4.14. Statistical Analysis

The Sigma Plot program (SigmaPlot^®^ version 14.5, Jandel Corporation) was used for the statistical analysis. The data are presented as the mean ± standard error. Statistical significance was determined with Tukey’s one-way ANOVA and a post hoc test. A *p* ≤ 0.05 was considered as significant.

## 5. Conclusions

The treatment with the HSL infusion at 2% improves the physiological renal function and anatomic structure of the kidneys in the rats with MS, through the antioxidant molecules that it possesses. These compounds inhibit pathways that metabolize the AA such as PLA_2_, COX2, 5-LOX, TAXS, and CYP450, but favor the COX1 pathway. This improves the vascular resistance of the renal arterioles, which increases the glomerular filtration rate, through the synthesis of prostaglandins.

## 6. Study Limitations

This study is a basic investigation where an HSL infusion at 2% was provided to a rat model that shows several characteristics of metabolic syndrome. The model was generated with consumption of 30% sucrose. Although the results in this study are clear, they may not be predictive for humans. In this work, we did not quantify the consumption of food for rodents, but in previous works already reported by our group, there is a significant difference in this regard between MS and C rats [44]. We did not assess the physical activity of the experimental animals either. On the other hand, we previously evaluated the increase in the activity of some antioxidant enzymes in the plasma of patients with Marfan syndrome before and after consumption, for 2 months, of an infusion of HSL at 2% with very promising results. Therefore, the current dosage has been previously implemented for human use [34]. In addition, in this previous study, none of the patients showed displeasure for the infusion since it had excellent palatability. Furthermore, infusions of HSL at a similar dose are commonly consumed with food in different countries, including Mexico. Therefore, the results from the present study support that the 2% HLS infusion could be used as an adjuvant therapy in the pathologies that comprise MS.

## Figures and Tables

**Table 1:** General characteristic of the rats in the experimental groups.

Variables	C	MS	MS+HSL	C+HSL
Glucose (mmol/L)	99.27 ± 5.59	161.85 ± 37.07	132.85 ± 7.29	110.14 ± 5.58
Insulin (μU/mL)	5.70 ± 0.75	20.33 ± 1.68 **	11.16 ± 1.21 *	4.89 ± 0.39
HOMA index	1.49 ± 0.12	15.08 ± 1.7 **	5.02 ± 0.32 *	1.40 ± 0.11
Triglycerides (mg/dL)	48.85 ± 4.84	187.42 ± 45.77 **	89.42 ± 4.23 *	49.57 ± 8.36
Cholesterol (mg/dL)	47.85 ± 8.05	46.71 ± 10.73	50.71 ± 1.97	43.71 ± 5.67
Intra-abdominal fat (g)	3.4 ± 0.3	11.6 ± 3.3 **	7.1 ± 0.8 *	3.8 ± 0.5
Systolic blood pressure (mmHg)	118.1 ± 5.7	141.2 ± 6.9 *	132.1 ± 4.1 *	117.63 ± 8.7

Data are mean ± SE; *n* = 6 for each group. Statistically significant at * *p* < 0.04, MS vs. MS+HSL; ** *p* = 0.001, C vs. MS. Abbreviations: C = control; MS = metabolic syndrome; MS+HSL = metabolic syndrome plus *Hibiscus sabdariffa* L.; C+HSL = control plus *Hibiscus sabdariffa* L.

**Table 2:** Renal function indicators in the rats of the experimental groups.

Variables	C	MS	MS+HSL	C+HSL
Drinking water (mL/24 h)	20.52 ± 2.58	55.10 ± 5.91 **	40.83 ± 4.54 **^†^**	33.33 ± 4.77
Weight of the right kidney (g)	1.27 ± 0.03	1.44 ± 0.05	1.28 ± 0.05	1.22 ± 0.5
Urine (mL/24 h)	14.42 ± 2.77	26.00 ± 4.27 **	24.85 ± 2.95	18.71 ± 4.79
CCr (mL/min)	2.88 ± 0.37	1.36 ± 0.14 **	2.38 ± 0.42 *	2.89 ± 0.86
Albuminuria (mg/mL)	38.30 ± 4.90	82.04 ± 9.21 **	49.54 ± 7.99 **^†^**	46.74 ± 4.18

Data are mean ± SE; *n* = 6 for each group. Statistically significant at * *p* ≤ 0.04, MS+HSL vs. MS; **^†^** *p* = 0.01, C vs. MS; ** *p* ≤ 0.003, C vs. MS. Abbreviations: C = control; MS = metabolic syndrome; MS+HSL = metabolic syndrome plus *Hibiscus sabdariffa* L.

**Table 3:** Prostaglandins in kidney homogenate of the experimental groups.

Variables	C	MS	MS+HSL	+HSL
PGE_2_ (pg/mL)	77.58 ± 0.02	77.77 ± 0.03 **	77.49 ± 0.02 **	77.61 ± 0.02
6-Keto-PGF_1α_ (pg/mL)	112.45 ± 0.75	93.63 ± 1.68 *	195.15 ± 1.21 *	182.76 ± 0.39
LKB_4_ (pg/mL)	735.45 ± 4.26	805.20 ± 41.76 ^†^	605.80 ± 59.01 *	819.15 ± 29.36
TxB_2_ (pg/mL)	149.48 ± 12.23	189.53 ± 12.52 *	109.44 ± 19.31 *	154.82 ± 39.05
AA (%)	14.22 ± 1.18	9.3 ± 0. 68 **	12.67 ± 1.24 *	13.67 ± 0.73

Data are mean ± SE; *n* = 6 for each group. Statistically significant at * *p* = 0.01, MS vs. MS+HSL; ** *p* = 0.001, C vs. MS; ^†^ *p* = 0.09, NS. Abbreviations: AA = Arachidonic acid; C = Control; MS = Metabolic syndrome; MS+HSL = Metabolic syndrome plus *Hibiscus sabdariffa* L.

## Data Availability

The data in our study are available from the corresponding authors upon reasonable request.
